# Monitoring Exposure to Ebola and Health of U.S. Military Personnel Deployed in Support of Ebola Control Efforts — Liberia, October 25, 2014–February 27, 2015

**Published:** 2015-07-03

**Authors:** Anthony P. Cardile, Clinton K. Murray, Christopher T. Littell, Neel J. Shah, Matthew N. Fandre, Dennis C. Drinkwater, Brian P. Markelz, Todd J. Vento

**Affiliations:** 1 U.S. Joint Forces Command, Operation United Assistance, Monrovia, Liberia; 2 Brooke Army Medical Center, Joint Base San Antonio, Fort Sam Houston, Texas

In response to the unprecedented Ebola virus disease (Ebola) outbreak in West Africa, the U.S. government deployed approximately 2,500 military personnel to support the government of Liberia. Their primary missions were to construct Ebola treatment units (ETUs), train health care workers to staff ETUs, and provide laboratory testing capacity for Ebola. Service members were explicitly prohibited from engaging in activities that could result in close contact with an Ebola-infected patient or coming in contact with the remains of persons who had died from unknown causes. Military units performed twice-daily monitoring of temperature and review of exposures and symptoms (“unit monitoring”) on all persons throughout deployment, exit screening at the time of departure from Liberia, and post-deployment monitoring for 21 days at segregated, controlled monitoring areas on U.S. military installations. A total of 32 persons developed a fever during deployment from October 25, 2014, through February 27, 2015; none had a known Ebola exposure or developed Ebola infection. Monitoring of all deployed service members revealed no Ebola exposures or infections. Given their activity restrictions and comprehensive monitoring while deployed to Liberia, U.S. military personnel constitute a unique population with a lower risk for Ebola exposure compared with those working in the country without such measures.

## Background

The Ebola epidemic in West Africa has caused approximately 11,000 deaths in Sierra Leone, Liberia, and Guinea (January 5, 2014–May 27, 2015) (1). The U.S. military deployed approximately 2,500 service members to construct ETUs, conduct World Health Organization–based training of Liberian and international health care workers to staff the units, establish laboratories for Ebola testing, and deliver sustainable logistical ETU support.

CDC, the U.S. Department of Defense (DoD), and other agencies established exposure risk categories and clinical criteria to guide public health actions for potentially exposed or infected persons traveling from Ebola-affected countries (2–6). Risk categories for deployed DoD personnel differed from CDC categories for civilian populations (Table 1). From October 25, 2014, through February 27, 2015, the 101st Airborne Division (Air Assault) commanded military forces under Operation United Assistance. Monitoring and surveillance data from DoD personnel deployed to Liberia during this period were analyzed to evaluate the effectiveness of activity restrictions and unit monitoring in identifying potential Ebola exposures, and to describe the types of illnesses that occurred among deployed DoD personnel who developed fever.

## DoD Disease Monitoring and Screening Procedures

U.S. military units in Liberia conducted unit monitoring twice daily on all deployed service members (2). Any person with a temperature ≥100.4°F (≥38.0°C), or any exposure or symptom concerns, was taken to the nearest DoD medical facility for evaluation by medical personnel. These personnel completed an Ebola risk assessment using a standard screening form (available at http://www.dtic.mil/whs/directives/forms/eforms/dd2990.pdf) (2). Service members’ adherence with prescribed malaria chemoprophylaxis also was assessed as part of the daily unit monitoring program. At locations where U.S. military units were based, Liberian government employees screened temperatures of all entering persons at controlled access points. Non-U.S. personnel with fever were denied entry, and febrile U.S. personnel were referred for on-site medical evaluation. Service members were prohibited through military orders from providing medical care to local nationals, being in close proximity to or having physical contact with any person known to have Ebola, eating local food including “bush-meat,” and having contact with the remains of persons who might have died from Ebola or whose cause of death was unknown.

Military public health authorities also monitored disease surveillance trends and febrile illness in deployed service members (Table 2). Final diagnoses were based on clinical assessment, because laboratory capabilities were limited to rapid diagnostic tests for malaria (BinaxNOW, Alere Inc.) and limited blood chemistry and hematology laboratory tests. Testing for Ebola virus by reverse transcription–polymerase chain reaction (RT-PCR) was available for patients with consistent signs and symptoms and an epidemiologic risk factor. Decisions about Ebola testing were made in consultation with U.S. military infectious disease and public health authorities deployed to Liberia.

Approximately 12 hours before departing Liberia, and after verification of compliance with unit monitoring during the preceding 21 days, medical providers screened departing service members for Ebola exposures, fever, and symptoms of possible Ebola, using a separate exit screening form (available at http://www.dtic.mil/whs/directives/forms/eforms/dd2991.pdf) (2). Upon returning to the United States, service members underwent controlled monitoring for 21 days at segregated locations on predesignated U.S. military installations.

## DoD Disease Monitoring and Screening Findings

The prevalence of illness among the deployed force averaged 1.8%, with gastrointestinal (33%), respiratory (22%), and dermatologic (20%) conditions accounting for the highest proportions of diagnoses. Thirty-two service members with febrile illness were identified (Table 2), representing 1% of all clinic visits and an estimated febrile illness rate of one case per 9,100 person-days in Liberia (estimated exposure time in Liberia for 2,540 service members was approximately 290,000 person-days, with mean duration of deployment of 110 days). The median time from date of country arrival to fever onset was 30 days (interquartile range = 14–50 days). Twenty (63%) persons reported being within 3 feet of a non-U.S. military person; none reported being within 3 feet of a known ill person or having direct contact with an ill person’s skin, blood, or body fluids. Fourteen (44%) febrile patients had never left their access-controlled facility since arriving in Liberia, and five (16%) persons with fever were detected through unit monitoring and unaware that they had fever. None of the 17 (53%) patients with fever and three or more Ebola-compatible symptoms had a close contact with an ill person. After receiving medical care and resolution of fever and symptoms, all patients resumed twice-daily unit monitoring. No febrile patient had an epidemiologic risk factor for Ebola that warranted Ebola RT-PCR testing, although two patients were tested for other reasons (a specimen collection exercise and a medical evacuation requirement) (Table 2). All 32 patients with fever completed a minimum of 21 days of post-fever monitoring by medical personnel.

No deployed service member had contact with a known or suspected Ebola patient, and exit screening on 2,540 persons identified no Ebola exposures, fever, or Ebola symptoms at the time of departure. After completion of an additional 21 days of twice-daily monitoring at controlled monitoring areas in the United States, no Ebola infections were identified.

## Discussion

Having been in a country with widespread transmission, deployed service members would be categorized, by CDC criteria, as low (but not zero) risk upon return to the United States. However, based on their non-Ebola care mission and stringent activity restrictions while deployed, they might be at lower risk for exposure than returning U.S. travelers who spent time in Liberia without such restrictions. A comparable assessment of an employer-directed program that actively monitored persons while they worked in an Ebola-affected country has not been published. A report of U.S. airport entry screenings of 1,993 travelers from Ebola-affected countries found that 86 (4%) were referred to CDC public health officers for medical evaluation, seven developed Ebola-compatible symptoms, and none had Ebola (7). This report supports observations that without close contact with an Ebola-infected patient, travel to an Ebola-affected country alone does not place a person at higher risk for Ebola infection.

An advantage of twice-daily monitoring in this deployed setting was that exposure assessments were less likely to be subject to recall bias. In addition, enforced military orders compelling adherence to activity restrictions ensured compliance with the monitoring program. Civilian employers might not have the same capacity to validate temperature, activity, exposure, and symptom history over an extended period of service in an Ebola-affected country. A further benefit of twice-daily symptom and temperature monitoring is that in the event of an Ebola exposure, an infection would be detected early, permitting expedited isolation and more timely treatment and medical evacuation.

Although the precautions put into place to prevent Ebola exposures appear effective, a full assessment of the effectiveness of the monitoring program for Ebola disease is not possible. The accuracy of the screening questionnaire might have been impacted by a respondent’s knowledge of a close contact’s clinical status. In addition, the potential for secondary gain from not telling the truth, such as avoiding isolation or quarantine, may underestimate exposure risk.

Health ministries in Ebola-affected countries, working directly with CDC and the World Health Organization, have established country exit screening and control measures, which include denying aircraft boarding to ill travelers and persons who report a high Ebola exposure risk (7). Knowledge of the activity restrictions and comprehensive monitoring of deployed U.S. military personnel might better inform clinical decision-making for returning military personnel and increase general awareness for communities receiving them.

**Summary**

What is already known on this topic?

Health ministries in countries affected by Ebola virus disease (Ebola), working with CDC and the World Health Organization, have established country exit screening measures to limit the spread of Ebola, and CDC established guidance for monitoring and movement of persons entering the U.S. from Ebola-affected countries. A recent study of 1,993 airport entry screenings of U.S. travelers returning from Ebola-affected countries found that none developed Ebola, suggesting that travel alone does not increase risk for infection.

What is added by this report?

U.S. military personnel deployed to Liberia were subjected to strict activity restrictions and twice-daily monitoring for fever, exposure to Ebola, or Ebola symptoms. Among approximately 2,500 deployed personnel, 32 had a febrile illness, including five who were unaware of their fever. The most frequent diagnoses were gastrointestinal, respiratory, and dermatologic conditions. No febrile person had had contact with an Ebola patient; no documented Ebola exposures or infections occurred among U.S. service members while they were in Liberia or after returning to the United States.

What are the implications for public health practice?

U.S. military personnel constitute a unique population because of their activity restrictions and aggressive monitoring. Knowledge of these measures might better inform clinical decision-making for these returning U.S. travelers and increase public awareness about their low exposure risk.

## Figures and Tables

**TABLE 1:** Summary of CDC and U.S. Department of Defense Ebola virus disease (Ebola) exposure risk categories

Exposure category	U.S. Department of Defense (October 10 and 31, 2014)	CDC (December 24, 2014)
**High risk**	Percutaneous (e.g., needle stick) or mucous membrane exposure to blood or body fluids of an Ebola patient	Percutaneous (e.g., needle stick) or mucous membrane exposure to blood or body fluids (including but not limited to feces, saliva, sweat, urine, vomit, and semen) from a person with Ebola while the person was symptomatic
	Direct skin contact to blood/body fluids	Direct contact without appropriate PPE with a person with Ebola while the person was symptomatic or the person’s body fluids
	Processing blood/body fluids of an Ebola patient without standard biosafety precautions	Laboratory processing of blood or body fluids from a person with Ebola while the person was symptomatic without appropriate PPE or standard biosafety precautions
	Direct contact with a dead body	Direct contact with a dead body without appropriate PPE in a country with widespread transmission or a country with cases in urban settings with uncertain control measures
		In countries with widespread transmission, having provided direct care in a household setting to a person with Ebola while the person was symptomatic
**Some risk**	Brief direct contact (e.g., shaking hands) with an Ebola patient	Direct contact while using appropriate PPE with a person with Ebola while the person was symptomatic or the person’s body fluids or being in the patient-care area of an Ebola treatment unit
		Any direct patient care in non-Ebola health care settings
	Household contact with an Ebola patient	Close contact in households, health care facilities, or community settings with a person with Ebola while the person was symptomatic
	Close contact (within 3 feet [1 meter] of an Ebola patient)	Close contact is defined as being within approximately 3 feet (1 meter) of a person with Ebola while the person was symptomatic for a prolonged period while not using appropriate PPE
	Prolonged period in an Ebola patient-care area	
**No known exposure**	Not in the some-risk or high-risk exposure category	NA
**Low (but not zero) risk**	NA	Having been in a country with widespread transmission, a country with cases in urban settings with uncertain control measures, or a country with former widespread transmission and now established control measures and having had no known exposures
		Brief direct contact (e.g., shaking hands), while not using appropriate PPE, with a person with Ebola while the person was in the early stage of disease
		Brief proximity with a person with Ebola while the person was symptomatic, such as being in the same room (not the patient-care area of an Ebola treatment unit) for a brief period
		In countries other than those with widespread transmission, direct contact while using appropriate PPE with a person with Ebola while the person was symptomatic or the person’s body fluids or being in the patient-care area of an Ebola treatment unit
		Laboratory processing of blood or body fluids from a person with Ebola while the person was symptomatic while using appropriate PPE and standard biosafety precautions
		Having traveled on an airplane with a person with Ebola while the person was symptomatic and having had no identified some-risk or high-risk exposures
**No identifiable risk**	NA	Laboratory processing Ebola-containing specimens in a biosafety level 4 facility
		Any contact with an asymptomatic person who had potential exposure to Ebola virus
		Contact with a person with Ebola before the person developed symptoms
		Any potential exposure to Ebola virus that occurred more than 21 days previously
		Having been in a country with Ebola cases but without widespread transmission, cases in urban settings with uncertain control measures, or former widespread transmission and now established control measures, and not having had any other exposures
		Having remained on or in the immediate vicinity of an aircraft or ship during the entire time that the aircraft or ship was in a country with widespread transmission or a country with cases in urban settings with uncertain control measures, and having had no direct contact with anyone from the community
		Having had laboratory-confirmed Ebola and subsequently been determined by public health authorities to no longer be infectious (i.e., Ebola survivors)

**Abbreviations:** NA = not applicable; PPE = personal protective equipment.

* Some-risk and high-risk exposure categories apply to persons who had the listed exposure during the preceding 21 days without wearing appropriate PPE.

**TABLE 2:** Demographic and clinical characteristics of U.S. military service members (N = 32) who developed febrile illness during Operation United Assistance — Liberia, October 25, 2014–February 27, 2015

Demographic or clinical characteristic	No.	(%)
**Male**	24	(75)
**Age in years, median (interquartile range)**	26	(25–36)
**Days in Liberia, median (interquartile range)**	30	(14–50)
**Having been within 3 feet of a non-U.S. military person during preceding 21 days** *	20	(63)
**Maximum temperature (°F), median (interquartile range)**	101.5	(101.0–102.7)
**Self-referral for medical evaluation**	27	(84)
**Referred by unit monitoring program for medical evaluation**	5	(16)
**DoD Ebola risk exposure category**		
No known exposure	32	(100)
Some risk for exposure	0	—
High risk for exposure	0	—
**Associated symptoms**		
Headache	17	(53)
Weakness	16	(50)
Myalgias	10	(31)
Arthralgias	6	(19)
Nausea	14	(44)
Vomiting	10	(31)
Diarrhea	18	(56)
Sore throat	4	(13)
Rigors/Chills	16	(52)
Cough	4	(13)
Rash	3	(9)
Back pain	6	(19)
Unexplained hemorrhage†	1	(3)
Confusion	1	(3)
Fever and ≥3 potential Ebola-compatible symptoms§	17	(53)
**Ebola virus RT-PCR result** ¶		
Positive	0	—
Negative	2	(6)
Malaria rapid diagnostic test** **result**		
Positive	0	—
Negative	26	(81)
**Clinical diagnosis**		
Viral syndrome	11	(34)
Gastroenteritis	12	(38)
Undifferentiated fever	6	(19)
Pharyngitis	1	(3)
Meningitis	1	(3)
Urinary tract infection	1	(3)
**Patient requiring medical evacuation from Liberia** ††	1	(3)
Patients with recurrent fever§§ and/or symptoms within 21 days	2	(6)

**Abbreviations:** DoD = U.S. Department of Defense; RT-PCR = reverse transcription–polymerase chain reaction.

* No service member reported close contact with an ill person, or contact with skin, blood, or body fluids of any person.

† Petechiae on soft palate and bilateral lower extremities.

§ DoD-defined potential Ebola symptoms include headache, myalgias, arthralgias, abdominal pain, vomiting, diarrhea, new skin rash, and unexplained bruising or bleeding.

¶ Ebola RT-PCR testing was not conducted because of epidemiologic risk: one was conducted as an initial test of system processes and response times; the other to fulfill an air evacuation requirement despite a non-Ebola illness.

** BinaxNOW (Alere Inc.).

†† One febrile patient, who also had no known Ebola exposure, was medically evacuated for meningitis (subsequently diagnosed with enterovirus infection by RT-PCR), recovered, and returned to full duty in Liberia.

§§ One person with recurrent fever was identified through twice-daily unit monitoring with a temperature of 101.9°F (38.8°C) after being afebrile for 96 hours, and was unaware of an elevated temperature. The patient had no associated symptoms, a normal physical examination, a negative BinaxNOW test for malaria, and resolution of fever within 24 hours. A second patient had a repeat episode of gastroenteritis that was successfully treated with azithromycin. Both recurrent fever patients had no known Ebola exposure. Neither patient had recurrence of fever after resuming daily unit monitoring for 21 days after the second fever episode.
